# Alpha1-antitrypsin protects the immature mouse brain following hypoxic-ischemic injury

**DOI:** 10.3389/fncel.2023.1137497

**Published:** 2023-03-06

**Authors:** Shan Zhang, Wendong Li, Yiran Xu, Tao Li, Joakim Ek, Xiaoli Zhang, Yafeng Wang, Juan Song, Changlian Zhu, Xiaoyang Wang

**Affiliations:** ^1^Henan Key Laboratory of Child Brain Injury and Henan Pediatric Clinical Research Center, Institute of Neuroscience and Third Affiliated Hospital of Zhengzhou University, Zhengzhou, China; ^2^Center for Brain Repair and Rehabilitation, Institute of Neuroscience and Physiology, University of Gothenburg, Gothenburg, Sweden; ^3^Henan Children’s Neurodevelopment Engineering Research Center, Children’s Hospital Affiliated to Zhengzhou University, Zhengzhou, China; ^4^Centre of Perinatal Medicine and Health, Institute of Neuroscience and Physiology, University of Gothenburg, Gothenburg, Sweden; ^5^Department of Women’s and Children’s Health, Karolinska Institutet, Stockholm, Sweden; ^6^Centre of Perinatal Medicine and Health, Institute of Clinical Science, University of Gothenburg, Gothenburg, Sweden

**Keywords:** alpha1-antitrypsin, neuroprotection, hypoxia-ischemia, neonatal brain injury, immature brain, motor dysfunction

## Abstract

**Introduction:** Preterm brain injury often leads to lifelong disabilities affecting both cognitive and motor functions, and effective therapies are limited. Alpha1-antitrypsin (AAT), an endogenous inhibitor of serine proteinases with anti-inflammatory, anti-apoptotic, and cytoprotective properties, might be beneficial in treating preterm brain injury. The aim of this study was to investigate whether AAT has neuroprotective effects in a mouse preterm brain injury model.

**Methods:** Preterm brain injury was induced on postnatal day 5, and mouse pups’ right common carotid arteries were cut between two ligations followed by hypoxia induction. Brain injury was evaluated through immunohistochemistry staining and magnetic resonance imaging. Fluoro-Jade B and immunohistochemistry staining were performed to investigate the neuronal cell death and blood-brain barrier (BBB) permeability. The motor function and anxiety-like behaviors were revealed by CatWalk gait analysis and the open field test.

**Results:** After hypoxia-ischemia (HI) insult, brain injury was alleviated by AAT treatment, and this was accompanied by reduced BBB permeability, reduced neuronal cell death and caspase-3 activation, and inhibition of microglia activation. In addition, AAT administration significantly improved HI-induced motor function deficiencies in mice. The neuroprotective effect of AAT was more pronounced in male mice.

**Conclusion:** AAT treatment is neuroprotective against preterm brain injury in neonatal mice, and the effect is more pronounced in males.

## Introduction

Preterm birth is a major global health problem in terms of neonatal morbidity and mortality (Perin et al., 2022). Improvements in perinatal medicine and neonatal intensive care have increased preterm infant survival, but survivors are at high risk for neonatal morbidities such as brain injury (Song et al., 2016; Juul et al., 2020; Liu et al., 2020). Preterm brain injury is mainly manifested as white-matter injury or intraventricular hemorrhage and is a leading cause of neurodevelopmental disabilities such as cerebral palsy (CP), intellectual disability, autism spectrum disorders, deafness, and blindness (Hafström et al., 2018; Ballabh and de Vries, 2021; Song et al., 2021). The pathogenesis of preterm brain injury is multifactorial, and both infection/inflammation and hypoxia-ischemia (HI) are thought to play crucial roles by inducing oxidative stress and neuroinflammation and subsequent neural cell death and by inhibiting pre-oligodendrocyte maturation (Hagberg et al., 2015; van Tilborg et al., 2018). Growing evidence shows that there is a tertiary phase of ongoing inflammation and neural cell death for months after the initial brain injury, which interrupts brain repair and functional development and contributes to neurological sequelae (Fleiss and Gressens, 2012; Zhang et al., 2017). There is currently no widely accepted therapeutic strategy to prevent or treat preterm brain injury, although several preclinical and clinical studies have shown the neuroprotective effects or reduced incidence of neurological disability in preterm infants with the use of recombinant human erythropoietin and stem cell therapy (Song et al., 2016; Vaes et al., 2021; Yates et al., 2021; Wu et al., 2022). Supportive care to maintain the stability of vital signs is still the main treatment for preterm brain injury. There is thus a pressing need for improving our understanding of the mechanisms of perinatal brain injury and for conducting comparative and translational studies on how to reduce cell death, increase cell survival, and promote brain regeneration and repair after preterm brain injury.

Serine proteases appear to play important roles in cellular physiology, and endogenous serine protease inhibitors regulate the activity of the serine proteases in the cell. Both serine proteases and serine protease inhibitors have been implicated in the development, plasticity, and pathology of the nervous system (Yoshida and Shiosaka, 1999). The neuroprotective effects of certain serine protease inhibitors have been reported, raising questions about their potential role in treating brain injury (Zhang et al., 2022). Alpha1-antitrypsin (AAT) is an abundant serine protease inhibitor belonging to the serine protease superfamily (Hazari et al., 2017). AAT has been shown to have an anti-inflammatory function that modulates the inflammatory response by reducing caspase-1 activity (Toldo et al., 2011), inducing the production of the anti-inflammatory cytokine IL-10 (Janciauskiene et al., 2007), and suppressing microglia-mediated neuroinflammation (Gold et al., 2014; Zhou et al., 2018). In addition to its anti-inflammatory effect, AAT also has anti-apoptotic properties and has been shown to inhibit caspase-3 activity (Petrache et al., 2006) and to regulate apoptotic cell death in neurons and neutrophils (Bergin et al., 2014; Cabezas-Llobet et al., 2018). Furthermore, AAT plays a cytoprotective role in vascular endothelial cells to reduce ischemia-reperfusion-induced vascular injury (Feng et al., 2015). Although many studies on AAT therapy have focused on α1-antitrypsin deficiency-related lung disease (Miravitlles et al., 2017; McEnery et al., 2022), other studies have demonstrated that AAT is also effective in treating type 1 diabetes and ischemic stroke damage (Guttman et al., 2014; Moldthan et al., 2014). However, the role of AAT in preterm brain injury is unknown.

An important neuroanatomical feature of the brain injury observed in preterm infants is a failure in myelination, referred to as white matter injury in the preterm brain (Ophelders et al., 2020). In the central nervous system, myelin is formed by oligodendrocytes, which mature from premyelinating oligodendrocyte (preOLs; Khwaja and Volpe, 2008). Due to the large quantity of preOLs in the periventricular white matter in infants between gestational weeks 24 and 32, the neonatal human brain is particularly susceptible to HI during this period of development (Back et al., 2001). In rodents, preOLs are abundant from postnatal day 2 (P2) to P5 (Craig et al., 2003). Thus, in this study, we used the well-established neonatal HI mouse model adapted to P5 mice (Albertsson et al., 2014) to investigate whether AAT has neuroprotective effects against preterm brain injury.

## Materials and methods

### Animals and the hypoxic-ischemic brain injury model

C57BL/6J male and female mice aged 8–10 weeks were purchased from Janvier Labs (Paris, France) and housed in a temperature-controlled and pathogen-free environment with a 12:12-h light-dark cycle. The pups were generated by crossing female and male mice. P5 mouse pups were anesthetized with isoflurane (5% for induction, 1.5–2.0% for maintenance) in a mixture of air and oxygen (1:1), and the right common carotid artery was cut between double ligatures. After the surgical operation, pups were returned to their dams for 1 h. Later, pups were placed in a chamber perfused with a humidified gas mixture (10% oxygen in nitrogen) at 36°C for 45 min. After hypoxic exposure, the pups were returned to their dams until sacrifice or until weaning at P21. Pups that died or bled profusely during the surgical operation were excluded from study. A total of 12 pups were excluded, and a total of 170 pups were used in all experiments. The pups were arbitrarily assigned to each group stratified by sex. Pups for behavior tests were assigned to normal, vehicle-treated HI, and AAT-treated HI groups, and pups for other experiments were assigned to vehicle-treated HI and AAT-treated HI groups. The sample size was calculated based on experiences in our previous studies (Li et al., 2019; Rodriguez et al., 2020). Mice were maintained in the Laboratory for Experimental Biomedicine of Gothenburg University, and all of the experiments were performed in accordance with Swedish national guidelines established by the Swedish Board of Agriculture (SJVFS 2019: 9) and were approved by the Gothenburg Animal Ethics Committee (2200/2019).

### AAT administration

**Figure 1:**
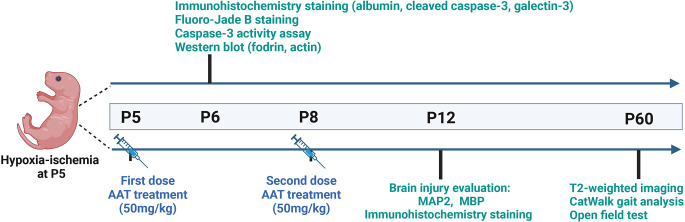
Experimental timeline.

The powdered AAT (Sigma, Cat# 9041-92-3) was dissolved in saline at 1 mg to 20 μl for the stock solution and then diluted five times in saline for the working solution. We performed a comparison between intranasal and intraperitoneal administration of AAT treatment and found that the concentration of AAT in the brain tissue was higher after intraperitoneal administration compared to intranasal administration (Supplementary Figure 1). Thus, AAT was administered intraperitoneally in our study. The treatment dose of AAT was chosen based on previous research found in the literature (Janciauskiene et al., 2011; Toldo et al., 2011, 2016; Lewis, 2012; Moldthan et al., 2014; Mauro et al., 2017) and our preliminary experiments. AAT treatment (50 mg/kg) was given twice, with the first dose being given immediately after HI at P5 and the second dose given 72 h later at P8. Vehicle control pups received the same volume of saline intraperitoneally. The whole experimental procedure is illustrated in Figure 1.

### Immunohistochemistry staining

Pups were deeply anesthetized with an overdose of sodium pentobarbital and perfused intracardially with PBS. The mouse brains were fixed in 5% buffered formaldehyde (Histofix; Histolab, Gothenburg, Sweden) at 4°C overnight. After dehydration with graded ethanol and xylene, brain samples were paraffin-embedded and cut into 5 μm coronal sections. Brain sections were deparaffinized in xylene and rehydrated in ethanol. Antigen retrieval was performed by boiling sections in sodium citrate buffer, and nonspecific binding was blocked with 4% donkey serum in PBS. The primary antibodies were mouse anti-microtubule-associated protein 2 (MAP2; 1:1,000 dilution, clone HM-2, Sigma, M4403), mouse anti-myelin basic protein (MBP; 1:500 dilution, clone SMI94, BioLegend, 836504), rabbit anti-cleaved caspase-3 (1:200 dilution, ASP175, Cell Signaling Technology, Beverly, Cat# 9661), goat anti-albumin (1:6,000 dilution, Abcam, Cambridge, UK, Cat# ab19194), and rat anti-galectin-3 (1:200 dilution, Invitrogen, Carlsbad, CA, USA, Cat# 14-5301-82). After primary antibody incubation, the appropriate biotinylated secondary antibodies (1:200 dilutions; all from Vector Laboratories, Burlingame, CA, USA) were added for 60 min at room temperature. After blocking endogenous peroxidase activity with 3% H_2_O_2_, sections were visualized with Vectastain ABC Elite (Vector Laboratories) and 0.5 mg/ml 3,3’-diaminobenzidine enhanced with ammonium nickel sulfate, β-D glucose, ammonium chloride, and β-glucose oxidase.

### Fluoro-jade B staining

After deparaffinization, sections were incubated with freshly prepared 0.06% potassium permanganate (KMnO_4_) for 15 min and rinsed in distilled water. Slides were then incubated with 0.0004% Fluoro-Jade B (Merck Millipore, Burlington, USA, Cat# AG310-30MG) in 0.09% acetic acid for 30 min in a dark container. After rinsing with distilled water, slides were dehydrated and covered with mounting medium.

### Brain injury evaluation

Paraffin-embedded brain samples were cut into 5 μm thick coronal sections, and eight consecutive coronal sections with an interval of 500 μm between each section were measured for each brain. The gray-matter area was determined by measuring the MAP2 immunoreactive area, and the subcortical white matter area was determined by measuring the MBP immunoreactive area. The MAP2-positive and MBP-positive areas in each section were measured in both hemispheres, and the volume was calculated using the following formula: V = ΣA × P × T, where V = the total volume, ΣA = the sum of area measurements, P = the inverse of the sampling fraction, and T = the section thickness. The total tissue loss volume ratio of gray matter and subcortical white matter was calculated using the following formula: [(contralateral hemisphere MAP-2 or MBP-positive volume − ipsilateral hemisphere MAP-2 or MBP-positive volume) / contralateral hemisphere MAP-2 or MBP-positive volume]. All of the evaluations were performed using Image J software by investigators blinded to group assignment.

### Cell counting

Area contours with fixed locations were drawn and measured in every 50th section. The gelactin-3-positive and caspase-3-positive cells were counted within a defined area (one visual field) of the cortex (100×), striatum (100×), CA1 (100×), and habenular nuclei (200×) for each section. The Fluoro-Jade B-positive cells were counted within the same area of the cortex (100×), striatum (100×), CA1 (200×), and habenular nuclei (200×) for each section. All the evaluations were performed using Image J software by investigators blinded to group assignment.

### Protein extraction and immunoblotting

Brain tissue from the parietal cortex in both hemispheres was dissected out and homogenized immediately on ice with a Dounce tissue homogenizer (Sigma, D8938) in tissue lysis buffer [15 mM Tris-HCl, pH 7.6, 320 mM sucrose, 1 mM dithiothreitol, 1 mM MgCl_2_, 3 mM EDTA-K, and 0.5% protease inhibitor cocktail (Sigma, P8340)]. The homogenate was centrifuged at 4°C and 9,200× *g* for 15 min, and the protein concentration of the supernatant was measured using the bicinchoninic acid method. Individual samples of 20 μg protein were loaded and run on 4%–12% NuPAGE Bis-Tris gels (Invitrogen, Cat# NP0336BOX) then transferred to reinforced nitrocellulose membranes (Bio-Rad, Cat# 162-0112). Membranes were incubated with mouse anti-fodrin (1:1,000 dilution, Enzo Life Sciences, Cat# BML-FG6090-0500) and rabbit anti-actin (1:200 dilution, Sigma, Cat# A2066) overnight at 4°C. After washing, the membranes were incubated with peroxidase-labeled goat anti-rabbit IgG antibody (1:2,000 dilution, Vector, Cat# PI-1000) or peroxidase-labeled horse anti-mouse IgG antibody (1:4,000 dilution, Vector, Cat# PI-2000). Immunoreactive species were visualized using the Super Signal West Pico PLUS Chemiluminescent Substrate (ThermoFisher Scientific, Cat# 34580) and a LAS 3000 cooled CCD camera (Fujifilm, Tokyo, Japan).

### Caspase-3 activity assay

A total of 25 μl homogenate sample was mixed with 75 μl extraction buffer containing 50 mM Tris-HCl (pH 7.3), 100 mM NaCl, 5 mM EDTA, 1 mM EGTA, 1 mM PMSF, and 1% protease inhibitor cocktail on a microtiter plate. After incubation for 15 min at room temperature, 25 μM caspase-3-substrate (Ac-DEVD-AMC, Peptide Institute, 670613) in 100 μl assay buffer was added. Caspase-3 activity was measured using a Spectramax Gemini microplate fluorometer (excitation/emission wavelength 380/460 nm every 2 min for 1 h at 37°C) and expressed as pmol AMC/mg protein per minute.

### T2-weighted imaging

T2-weighted imaging was performed on a preclinical MR scanner 4.7 T (MR Solution, United Kingdom) at P60. Fast spin echo sequence was used for T2-weighted imaging, and the scanning parameters were set as follows: repetition time = 5,000 ms, echo time = 51 ms, reversal angle = 180°, field of view = 22 mm × 22 mm, matrix = 256 × 256, number of slices = 18, slice thickness = 1.0 mm. Preclinical Scan 1.2 software was used for image acquisition, and the volume and statistics functions in the ITK-SNAP 3.8.0 software was used for tissue volume quantification. The total tissue loss volume was calculated as the contralateral hemisphere volume minus the ipsilateral hemisphere volume.

#### CatWalk XT gait analysis

The CatWalk XT is a gait analysis system with a 1.3 m horizontal glass gate covered by a removable tunnel creating a dimmed light on the walkway. The mice were trained to walk at the beginning of the walkway and to traverse the plate towards their home cage voluntarily. Data were collected by a high-speed color camera located beneath the glass gate and sent to a connected computer. Data analysis was performed automatically by the CatWalk XT software. After successive trainings for 5 days, mice performed a minimum of three nonstop runs for quantification in the CatWalk XT analysis software. A maximum speed variation of 60%, a camera gain of 28.7 dB, and a detection threshold of 0.1 were set for the detection of all parameters used in the experiments.

#### Open field test

The open field test was conducted in a black acrylic glass box (100 cm × 100 cm × 40 cm) with an overhead lamp pointed at the center of the field. Each mouse was placed in the corner of the apparatus, and locomotion parameters were recorded for 5 min. All mice underwent a 30-min acclimation period prior to the start of open field test.

#### Statistical analysis

The normality of all data was tested by the Shapiro–Wilk test, and the homogeneity of variance of all data was tested by Levene’s test. For comparisons between two groups, unpaired *t*-tests were used for data with normal distribution and homogeneity of variance, and the Mann–Whitney U-test was applied for data with a non-normal distribution. For comparisons between three groups, one-way ANOVA with a Bonferroni *post-hoc* test was used for data with normal distribution and homogeneity of variance, and the Kruskal–Wallis test was used for data with a non-normal distribution. For two-dimensional data, two-way ANOVA with Bonferroni *post-hoc* test was used for data with normal distribution and homogeneity of variance, and the Scheirer–Ray–Hare test was used for data with a non-normal distribution. The results are presented as means ± standard deviations, and *p* < 0.05 was considered statistically significant. IBM SPSS 21.0 (NY, USA) was used for data analysis.

## Results

### AAT treatment alleviated brain injury after HI

**Figure 2:**
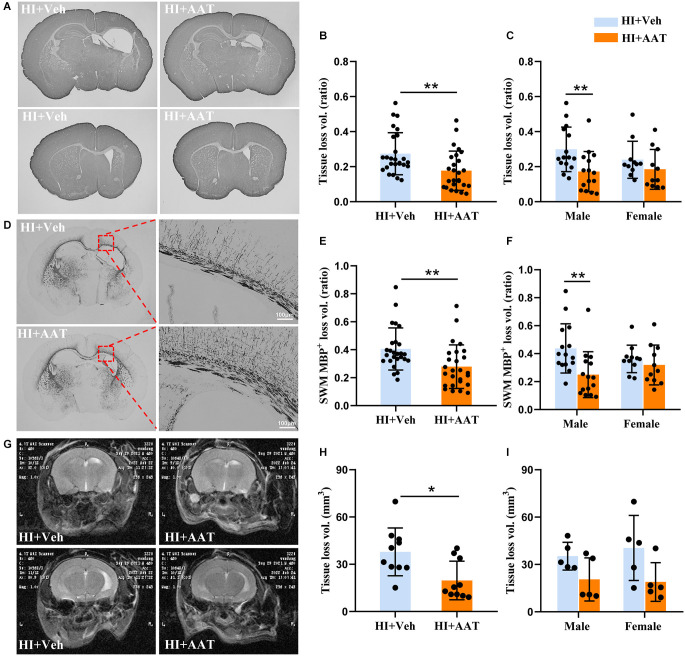
AAT treatment reduced HI-induced neonatal mouse brain injury. **(A)** Representative MAP2 staining of coronal brain sections at the hippocampus level (upper panels) and striatum level (lower panels) at P12 after HI in the vehicle-treated and AAT-treated groups. **(B)** Quantification of total brain tissue volume loss ratio at P12 (*n* = 26/group, *p* = 0.003). **(C)** Quantification of total brain tissue volume loss ratio at P12 in male and female mice (*n* = 15/group for males, *p* = 0.004; *n* = 11/group for females, *p* = 0.277). **(D)** Representative MBP staining at the hippocampus level showing the myelin structure in the subcortical white matter at P12 after HI in the vehicle-treated and AAT-treated groups. The right panels show higher magnifications of the MBP-stained subcortical white matter in the ipsilateral hemisphere. **(E)** Quantification of the volume loss of MBP^**+**^ subcortical white matter at P12 (*n* = 26/group, *p* = 0.002). **(F)** Quantification of the volume loss of MBP^**+**^ subcortical white matter at P12 in male and female mice (*n* = 15/group for males, *p* = 0.001; *n* = 11/group for females, *p* = 0.505). **(G)** Representative cerebral coronal T2-weighted images at P60 after HI in vehicle-treated and AAT-treated groups. **(H)** Quantification of total brain tissue volume loss at P60 (*n* = 10/group, *p* = 0.011). **(I)** Quantification of total brain tissue volume loss at P60 in male and female mice (*n* = 5/group for males, *p* = 0.222; *n* = 5/group for females, *p* = 0.079). *p < 0.05, **p < 0.01.

We first wanted to examine whether AAT treatment affected HI-induced brain damage. Brain injury was evaluated by measuring the total tissue loss volume ratio at 7 days after HI based on MAP2 immunochemistry staining (Figure 2A). The average value in AAT-treated mice was 35.2% lower compared to vehicle-treated littermates (Figure 2B). However, a significant difference between AAT-treated and vehicle-treated mice was seen in males but not in females (Figure 2C). The average value was reduced by 42.5% in AAT-treated male mice compared to vehicle-treated male littermates, while the average value was only 22.8% lower in AAT-treated female mice compared to vehicle-treated female littermates (Figure 2C).

For evaluating the effect of AAT treatment on white matter, MBP immunochemistry staining was performed at 7 days after HI (Figure 2D). The white matter injury was calculated as the MBP-positive tissue loss volume ratio in the subcortical white-matter area. The average value in AAT-treated mice was decreased by 31.2% compared to vehicle-treated littermates (Figure 2E). Analyzing the data according to sex, we found again that AAT treatment significantly reduced the degree of white-matter injury in males but not in females, and the average value was 42.9% lower in AAT-treated male mice compared to vehicle-treated male littermates, but there was no significant difference in females between the AAT and vehicle groups (Figure 2F).

We also evaluated the long-term effect of AAT treatment on brain injury in neonatal mice by magnetic resonance imaging (Figure 2G). According to the results of T2-weighted imaging analysis at P60 (55 days after HI), there was a significant 47.9% reduction in the total tissue loss volume in the AAT-treated group compared to the vehicle-treated group (Figure 2H), but no sex difference was observed (Figure 2I).

### AAT treatment improved motor function deficiency but not anxiety-like behavior after HI

**Figure 3:**
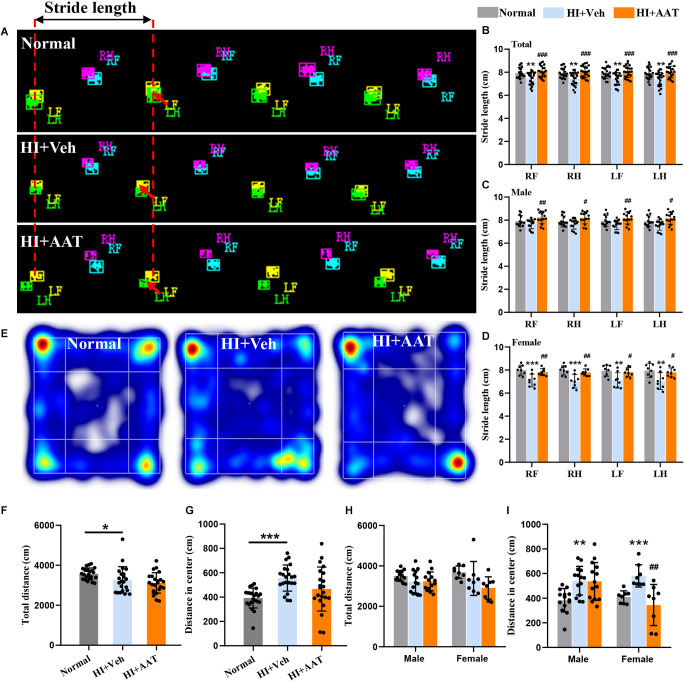
Motor function deficiency and anxiety-like behaviors in neonatal HI mice after AAT treatment. **(A)** Graphical representation of stride length in the normal, vehicle-treated, and AAT-treated groups. RF: right front paw; RH: right hind paw; LF: left front paw; LH: left hind paw. **(B)** Analysis of stride length of four paws in three groups [*n* = 22/group, normal *vs.* veh (*), RF (*p* = 0.007), RH (*p* = 0.006), LF (*p* = 0.005), LH (*p* = 0.006); veh *vs.* AAT (**#**), RF (*p* = 0.000), RH (*p* = 0.000), LF (*p* = 0.000), LH (*p* = 0.001)].** (C)** Analysis of stride length of four paws in male mice [*n* = 14/group, normal *vs.* veh (*), RF (*p* = 0.538), RH (*p* = 0.592), LF (*p* = 0.25), LH (*p* = 0.353); veh *vs.* AAT (**#**), RF (*p* = 0.007), RH (*p* = 0.01), LF (*p* = 0.007), LH (*p* = 0.015)]. **(D)** Analysis of stride length of four paws in female mice [*n* = 8/group, normal *vs.* veh (*), RF (*p* = 0.001), RH (*p* = 0.000), LF (*p* = 0.004), LH (*p* = 0.003); veh *vs.* AAT (**#**), RF (*p* = 0.009), RH (*p* = 0.006), LF (*p* = 0.017), LH (*p* = 0.018)]. **(E)** Representative heat map of locomotion in normal, vehicle-treated, and AAT-treated mice. **(F)** Analysis of the total distance in the open field in the three groups (*n* = 22/group, normal *vs.* veh (*), *p* = 0.018; veh *vs.* AAT (**#**), *p* = 0.589). **(G)** Analysis of distance traveled in the center area in the three groups (*n* = 22/group, normal *vs.* veh (*), *p* = 0.000; veh *vs.* AAT (**#**), *p* = 0.054). **(H)** Analysis of the total distance in the open field in male and female mice (*n* = 14/group for males, normal *vs.* veh (*), *p* = 0.397; veh *vs.* AAT (**#**), *p* = 1.000; *n* = 8/group for females, normal *vs.* veh (*), *p* = 0.676; veh *vs.* AAT (**#**), *p* = 0.22). **(I)** Analysis of distance traveled in the center area in male and female mice (*n* = 14/group, normal *vs.* veh (*), *p* = 0.005; veh *vs.* AAT (**#**), *p* = 1.000; *n* = 8/group for females, normal *vs.* veh (*), *p* = 0.001; veh *vs.* AAT (**#**), *p* = 0.003). *p < 0.05, **p < 0.01, ***p < 0.001; ^**#**^*p* < 0.05, ^**##**^*p* < 0.01, ^**###**^*p* < 0.001.

To determine whether AAT treatment affected HI-induced motor function deficiency and anxiety-like behavior in the mice, a battery of neurobehavior tests were performed at P60 (55 days after HI). Gait analysis by CatWalk XT showed that the paw parameter values of print area, maximum contact area, and mean intensity of all four paws were significantly reduced in the vehicle-treated group compared to normal mice, which indicated that motor development in neonatal mice was markedly impaired after HI injury. However, no significant differences in these parameters between vehicle-treated and AAT-treated groups were observed (Supplementary Figure 2). Stride length is defined as the distance between the placement of a paw and the subsequent placement of the same paw (Figure 3A), and the stride length of all four paws was significantly lower in vehicle-treated mice compared to normal mice, and this deficiency was rescued by AAT treatment (Figure 3B). We also analyzed changes in the stride length of all four paws between the AAT-treated and vehicle-treated groups by sex, and the protective effect of AAT administration was evident in both males and females (Figures 3C,D).

Anxiety-like behavior and locomotor activity were examined by the open field test (Figure 3E). The open field test of locomotor activity requires normal motor skills and is suitable for the evaluation of anxiety level and the response to a novel environment. Some outcomes, such as time spent in the center and distance traveled in the center, likely gauge some aspects of emotionality, including anxiety. We found that the total distance traveled in the open field of vehicle-treated HI mice was reduced compared to normal mice (Figure 3F). In addition, normal mice would walk along the periphery in a new environment, but this phenomenon was not observed in vehicle-treated HI mice, and the distance traveled in the center area by vehicle-treated HI mice was increased compared to normal mice (Figure 3G). AAT treatment did not have an effect on either the total distance traveled in the open field (Figure 3F) or the distance traveled in the center area (Figure 3G). Analyzing the data according to sex, AAT administration had no apparent effect on the total distance traveled in the open field in either male or female HI mice (Figure 3H), but an effect of AAT treatment on the distance traveled in the center area was found in female HI mice (Figure 3I).

### AAT treatment decreased the permeability of the blood-brain barrier (BBB) and inhibited microglia activation after HI

**Figure 4:**
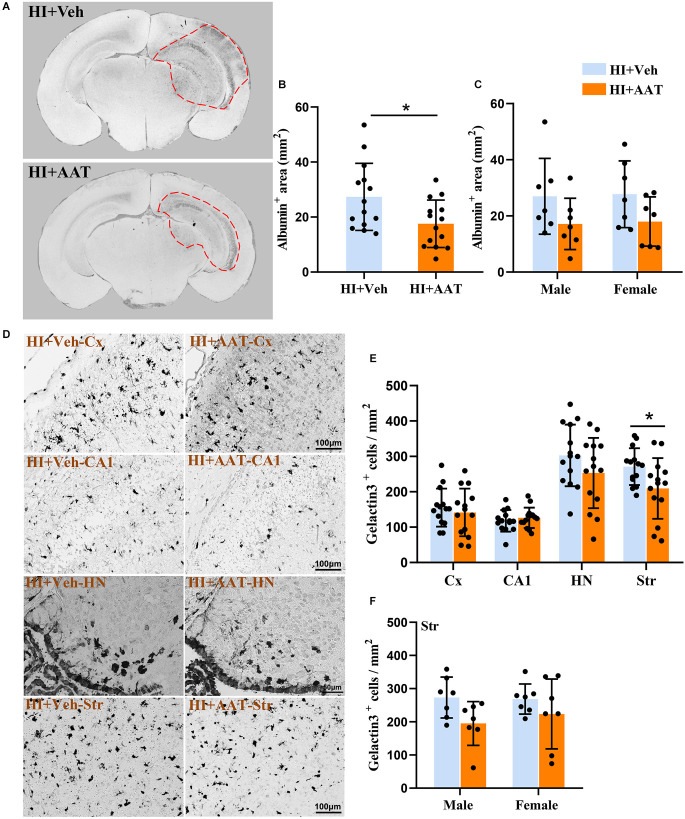
Albumin extravasation and microglia activation in neonatal HI mice after AAT treatment. **(A)** Representative pictures at the hippocampal level showing the albumin-positive areas in the neonatal mouse brain at 24 h after HI in vehicle-treated and AAT-treated groups. **(B)** Measurement of the albumin-positive area at 24 h after HI (*n* = 14/group, *p* = 0.022). **(C)** Measurement of the albumin-positive area at 24 h after HI in male and female mice (*n* = 7/group, *p* = 0.107 for males and *p* = 0.111 for females). **(D)** Representative images showing the immunochemistry staining of activated macroglia in the cortex (Cx), hippocampus (CA1), habenula nucleus (HN), and striatum (Str) at 24 h after HI in the vehicle-treated and AAT-treated groups. **(E)** Quantification of galectin-3-positive cells at 24 h after HI in the Cx, CA1, HN, and Str [*n* = 14/group, Cx (*p* = 0.508), CA1 (*p* = 0.464), HN (*p* = 0.172), Str (*p* = 0.03)]. **(F)** Quantification of galectin-3-positive cells in the striatum area at 24 h after HI in male and female mice (*n* = 7/group, *p* = 0.057 for males and *p* = 0.262 for females). *p < 0.05.

Extravagated albumin can be detected with immunohistochemical methods and is a straightforward way to demonstrate the presence of BBB leakage. We observed albumin staining in the parenchyma of the cortex, hippocampus, and part of the thalamic region in the ipsilateral hemispheres at 7 days after HI (Figure 4A). The albumin-positive area in the injured hemisphere was significantly reduced in AAT-treated mice compared to vehicle-treated mice (Figure 4B), but no sex difference was observed between the two groups (Figure 4C).

Galectin-3 immunochemistry staining was performed to detect the activation of microglia in brain tissue at 24 h after HI (Figure 4D). We measured the density of activated microglia cells in four brain regions (the cortex, CA1, habenular nuclei, and striatum), but only the density of activated microglia cells in the striatum was statistically reduced in AAT-treated vs. vehicle-treated mice (Figure 4E). Furthermore, the effects of AAT on microglial activation in the striatum did not exhibit any sex differences (Figure 4F).

### AAT treatment reduced neuronal cell death after HI

**Figure 5:**
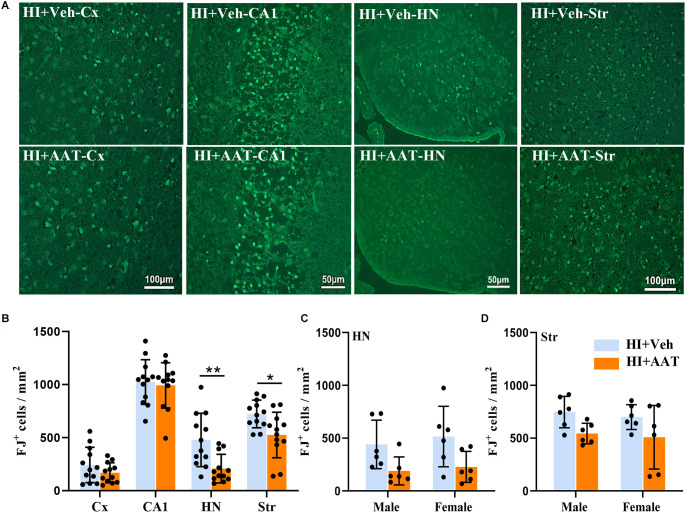
Neuronal cell death in neonatal HI mice after AAT treatment. **(A)** Representative Fluoro-Jade B staining in the cortex (Cx), hippocampus (CA1), habenula nucleus (HN), and striatum (Str) at 24 h after HI in vehicle-treated and AAT-treated groups. **(B)** Quantification of Fluoro-Jade B-labeled cells at 24 h after HI in the Cx, CA1, HN, and Str [*n* = 12/group, cortex (*p* = 0.192), CA1 (*p* = 0.719), HN (*p* = 0.005), Str (*p* = 0.012)]. **(C)** Quantification of Fluoro-Jade B-labeled cells in the habenula nucleus area at 24 h after HI in male and female mice (*n* = 6/group, *p* = 0.05 for males and *p* = 0.054 for females). **(D)** Quantification of Fluoro-Jade B-labeled cells in the striatum area at 24 h after HI in male and female mice (*n* = 6/group, *p* = 0.07 for males and *p* = 0.087 for females). *p < 0.05, **p < 0.01.

The quantification of neuronal cell death based on the number of Fluoro Jade B-positive cells demonstrated substantial overall neuronal cell loss at 24 h after HI (Figure 5A). Regional analysis showed that the numbers of dead or dying neuronal cells in the habenular nuclei and striatum were significantly reduced in AAT-treated compared to vehicle-treated mice (Figure 5B), but no sex difference was observed (Figures 5C,D).

### AAT treatment inhibited apoptosis after HI

**Figure 6:**
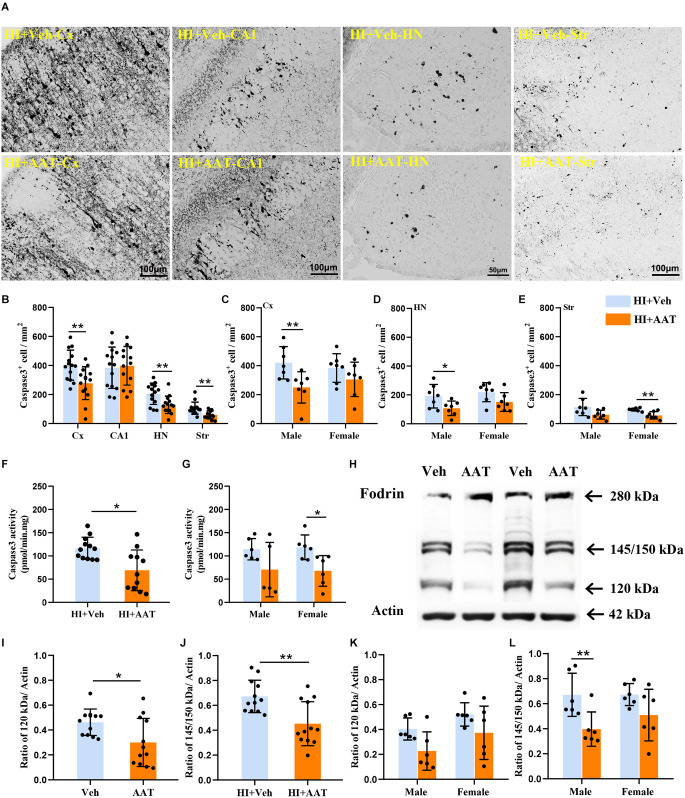
Activation of caspase-3 and calpain in neonatal HI mice after AAT treatment. **(A)** The photomicrographs show immunochemistry staining of activated caspase-3 in the cortex (Cx), hippocampus (CA1), habenula nucleus (HN), and striatum (Str) at 24 h after HI in the vehicle-treated and AAT-treated groups. **(B)** Quantification of cleaved caspase-3-labeled cells at 24 h after HI in the Cx, CA1, HN, and Str (*n* = 14/group, *p* = 0.005 in Cx, *p* = 0.437 in CA1, *p* = 0.006 in HN, *p* = 0.001 in Str). **(C)** Quantification of cleaved caspase-3-labeled cells in the cortex at 24 h after HI in male and female mice (*n* = 7/group, *p* = 0.008 for males and *p* = 0.193 for females). **(D)** Quantification of cleaved caspase-3-labeled cells in the habenula nucleus at 24 h after HI in male and female mice (*n* = 7/group, *p* = 0.028 for males and *p* = 0.067 for females). **(E)** Quantification of cleaved caspase-3-labeled cells in the striatum at 24 h after HI in male and female mice (*n* = 7/group, *p* = 0.059 for males and *p* = 0.006 for females). **(F)** The caspase-3 activity in cortical tissue homogenate was measured at 24 h after HI in the AAT-treated and vehicle-treated groups (Veh = 12, AAT = 11, *p* = 0.011). **(G)** Measurement of caspase-3 activity at 24 h after HI in male and female mice (Veh = 6, AAT = 5, *p* = 0.273 in male; *n* = 6/group, *p* = 0.015 in female). **(H)** Representative immunoblotting of fodrin and actin in the cortical tissue from the injured hemisphere of the vehicle and AAT treatment groups at 24 h after HI. **(I)** Quantification of 120 kDa fragment expression at 24 h after HI in vehicle-treated and AAT-treated groups (*n* = 12/group, *p* = 0.024). **(J)** Quantification of 145/150 kDa fragment expression at 24 h after HI in vehicle-treated and AAT-treated groups (*n* = 12/group, *p* = 0.002). **(K)** Quantification of 120 kDa fragment expression at 24 h after HI in male and female mice (*n* = 6/group, *p* = 0.051 for males and *p* = 0.097 for females). **(L)** Quantification of 145/150 kDa fragment expression at 24 h after HI in male and female mice (*n* = 6/group, *p* = 0.007 for males and *p* = 0.086 for females). *p < 0.05, **p < 0.01.

Caspase-dependent apoptotic cell death was investigated by measuring the active form of caspase-3 in different brain regions at 24 h after HI (Figure 6A). In the analyzed brain regions, caspase-3-positive cells were clearly increased in the cortex, habenular nuclei, and striatum in vehicle-treated mice compared to AAT-treated mice, but no significant changes were seen in the CA1 area between the two groups (Figure 6B). Caspase-3-positive cells were significantly decreased in AAT-treated male mice compared to vehicle-treated male mice in both the cortex and habenular nuclei (Figures 6C,D). However, in the striatum caspase-3 activation was significantly inhibited in AAT-treated female mice compared to vehicle-treated female mice (Figure 6E). Additionally, the caspase-3 activity assay clearly showed that AAT treatment significantly reduced caspase-3 activation after HI (Figure 6F), which was more pronounced in females (Figure 6G).

The 280 kDa non-erythroid fodrin protein is a widely studied substrate for calpain. The identification of fodrin cleavage fragments is a method to detect the activation of calpain and caspase-3 (Wang et al., 2001), and calpain-mediated proteolysis of fodrin results in 145 kDa and 150 kDa fragments while caspase-3-mediated cleavage results in a 120 kDa fragment (Figure 6H). From the Western blot quantification results, it was observed that the expression of both the 145/150 kDa fragments and 120 kDa fragment were significantly decreased in AAT-treated mice compared to vehicle-treated mice, which indicated that AAT treatment reduced the activation of both calpain and caspase-3 in the neonatal brain after HI injury (Figures 6I,J). The expression of proteolytically generated fodrin fragments between the two groups was also analyzed by sex, and the results showed that the inhibition of calpain activation by AAT administration was more pronounced in males (Figures 6K,L).

## Discussion

In this work we conducted a preclinical study to test the therapeutic efficacy of AAT in a mouse model of neonatal brain injury. We show for the first time that AAT treatment attenuates HI-induced preterm brain injury and improves motor function deficits and that the neuroprotective effect of AAT is more robust in males.

We found that AAT treatment attenuated both gray and white matter injury in an HI-induced preterm brain injury mouse model. Even though AAT is a 52 kDa protein, our results indicate that systemic administration AAT may cross BBB in the HI-injured immature brain, which could be related to the increased BBB permeability after cerebral HI insult (Ek et al., 2015). Improving neurofunctional outcome is one of the focuses of developing new intervention strategies for preterm brain injury. The stride length in HI mice after AAT treatment was improved significantly at P60. As a well-established parameter in the CatWalk XT analysis, an increase in the stride length after injury suggests greater trunk stability. To note, no other differences among the analysis parameters were observed between vehicle and AAT-treated mice, and one potential reason for this could be that we administered AAT only twice in a short period of time after HI, which may not have been long enough for AAT to give full protection and therefore may not reflect the effects of AAT on long-term neurodevelopment. In addition, the developing brain is highly neuroplastic, which may allow for rapid functional compensation, and thus the measurement of rodent functional defects should be performed in the early stage after brain injury (Ismail et al., 2017).

The open field test is used to analyze the general locomotor activity and anxiety-like behavior in rodents (Seibenhener and Wooten, 2015). The total distance traveled in the open field can reflect the baseline locomotion of mice in a more stressful condition. We observed that the baseline locomotion of HI mice in the open field was reduced compared to normal mice, and AAT administration did not show any apparent effect on the baseline locomotion of HI mice. Mice that prefer staying close to the walls and to travel more in the periphery can be described as showing thigmotaxis, which is more pronounced in mice showing signs of anxiety-like behavior (Lamprea et al., 2008). In the present study, it was found that the distance traveled in the center area by HI mice was increased, which is the exact opposite of the natural reflex of normal mice, indicating that mice had lower thigmotaxis and anxiety after HI. Although there was no significant difference in the distance traveled in the center area between vehicle-treated HI mice and AAT -treated HI mice, the distance traveled in the center area was significantly reduced in female mice of the AAT treated group, indicating that the thigmotaxis and anxiety level of AAT-treated HI female mice tended to be more consistent with those of normal mice. However, the mechanisms of these findings remain unclear and require further investigation.

Microglia play a pivotal role in perinatal brain injury, and they initially respond to stimuli such as HI or infection with the production of pro-inflammatory cytokines that eventually exacerbate brain injury (Hagberg et al., 2015). Microglia are associated with axonal damage and myelinating oligodendrocytes, which are major pathological components of white-matter injury (Shao et al., 2021), and it has been reported that suppressing the activation of microglia is one of the mechanisms for reducing HI-induced brain injury in neonatal mice (Arvin et al., 2002; Mallard et al., 2019). Our results showed that activated microglia in the striatum area were significantly reduced after AAT administration in HI mice. The BBB plays an important role in maintaining homeostasis and protecting neurons. The permeability of the BBB increases after HI insult in the neonatal mouse brain and persists for more than 24 h (Ek et al., 2015), and it is widely accepted that BBB dysfunction and microglia are closely related (Haruwaka et al., 2019). We found that BBB permeability was decreased after AAT treatment, and thus we speculate that AAT might attenuate brain injury by inhibiting microglial activation and decreasing BBB permeability during the initial pro-inflammatory phase of preterm brain injury following HI. However, the causal relationship between decreased BBB permeability, suppressed microglial activity, and AAT’s neuroprotective effect needs to be further explored.

The premature brain is susceptible to injury from infective, ischemic, and inflammatory insults (Vannucci and Hagberg, 2004; Eklind et al., 2005; Gussenhoven et al., 2018), and preOLs in the premature brain are particularly vulnerable to these insults due to limited antioxidant defense mechanisms and high levels of mitochondrial oxygen consumption (Buser et al., 2010; Spaas et al., 2021). In preterm brain injury, impaired preOLs negatively impact the maturation of oligodendrocytes and cause myelination failure (Buser et al., 2012; Motavaf and Piao, 2021). In addition to myelination disturbance, neuronal cell death is also involved in preterm brain injury. Current evidence suggests that apoptotic cell death plays a prominent role in preterm brain injury, especially the caspase-3-dependent apoptotic pathway (Truttmann et al., 2020). Apoptosis is a form of programmed cell death, and apoptosis inhibition reduces brain injury and improves neurological function in rodent models of cerebral ischemia (Han et al., 2002; Blomgren et al., 2007). Several caspase family members induce apoptosis and participate in the final execution phase of apoptosis, but caspase-3 appears to be an especially important effector enzyme in neuronal apoptosis (Porter and Jänicke, 1999; Broughton et al., 2009). We found that AAT treatment reduced caspase-3 activation after HI, and the neuroprotective effect of AAT treatment might be related to the inhibition of caspase-dependent apoptotic cell death.

Calpain is a calcium-dependent cysteine protease that has been proposed to participate in the turnover of cytoskeletal proteins and in the regulation of kinases, transcription factors, and receptors. Although calpain activation has historically been assumed to result in necrotic cell death, a contribution to apoptosis has also been suggested (Altznauer et al., 2004; Harwood et al., 2005). The expression of 145/150 kDa fodrin fragments in AAT-treated mice was significantly reduced, suggesting that the activation of calpain was decreased after AAT treatment, which is in line with previous studies. It has been shown that AAT modulates microglial-mediated neuroinflammation by inhibiting calpain activation *in vitro* (Gold et al., 2014), and calpain inhibitors have also been shown to have neuroprotective effects in perinatal brain injury (Blomgren et al., 1999; Kawamura et al., 2005). Altogether our results suggest that AAT exerts its neuroprotective effect mainly by reducing neuronal apoptotic cell death by inhibiting the activation of caspase-3 and calpain after HI injury in the immature brain. We previously showed that there is cross talk between caspase-3 and calpain activation (Blomgren et al., 2001), but whether AAT selectively inhibits caspase-3 or calpain alone or if it non-selectively inhibits both needs to be investigated further.

Sex-specific differences in the efficacy of certain neuroprotective drugs have been demonstrated in animal models of neonatal HI (Hagberg et al., 2004; Nijboer et al., 2007; Daher et al., 2018; Li et al., 2019; Rodriguez et al., 2020). Our previous study found that adaptaquin treatment reduced neonatal HI brain injury in a sex-dependent manner, and the neuroprotective effect was more obvious in males (Li et al., 2019), while another study reported the sex-dependent efficacy of magnesium sulfate in protecting against neonatal HI brain injury (Daher et al., 2018). In the present study, we found that the reduction in brain damage after AAT administration was more pronounced in male mice. It has been previously suggested that the sex differences in neuroprotection might be related to differences in the activation of neuronal cell death pathways after brain injury (Lang and McCullough, 2008), differences in sex hormones (Siddiqui et al., 2016), and differences in mitochondrial dysfunction (Demarest and McCarthy, 2015), but the underlying mechanisms behind the current observations need to be explored further.

There are some limitations in our study. First, the HI-induced preterm brain injury model does not fully represent preterm brain injury in infants. Currently, there is a lack of a perfect rodent model to best mimic brain injury in preterm infants. Although multiple animal models have been used to study preterm brain injury, such as IL-1β or LPS systemic injection-induced diffuse white matter injury in neonatal rodents (Favrais et al., 2011; Fan et al., 2013), ibotenic acid injection-induced white and gray matter injury in P5 mice (Sárközy et al., 2007), collagenase injection-induced intracranial hemorrhage in P5 rats (Jinnai et al., 2020), and HI-induced brain injury in P5 mice (Albertsson et al., 2014), none of them fully represent the pathology of brain injury seen in preterm infants. Second, the neuroprotective effect of AAT is more obvious in males according to the overall findings in our study, but the specific mechanisms underlying the sex differences in AAT neuroprotection are still unclear.

## Conclusion

AAT has neuroprotective effects in the immature brain following HI and thus may serve as a potential therapeutic strategy for preterm brain injury. The mechanisms underlying AAT’s neuroprotective effects in the immature brain warrant further investigation.

## Data availability statement

The original contributions presented in the study are included in the article/Supplementary material, further inquiries can be directed to the corresponding author/s.

## Ethics statement

The animal study was reviewed and approved by Gothenburg Animal Ethics Committee.

## Author contributions

CZ and XW conceived and designed the experiments. SZ, WL, TL, and YW performed the experiments. SZ, WL, YX, TL, XZ, and JE analyzed the data. SZ, YX, WL, XZ, CZ, and XW interpreted the results and prepared the figures. SZ, YX, CZ, and XW drafted the manuscript. JS, JE, CZ, and XW edited and revised the manuscript. The final manuscript was approved by all authors. All authors contributed to the article and approved the submitted version.

## Supplementary material

Supplementary Figure 1 AAT concentration in the brain by different administration routes. **(A)** AAT concentration in brain tissue at 3 h and 6 h after intranasal administration in the control and HI groups. **(B)** AAT concentration in brain tissue at 3 h and 6 h after intraperitoneal administration in the control and HI groups.

Supplementary Figure 2 CatWalk XT gait analysis. **(A)** Analysis of print area of the four paws in the normal, vehicle-treated, and AAT-treated groups [*n* = 22/group, normal *vs.* veh (*), RF (*p* = 0.000), RH (*p* = 0.000), LF (*p* = 0.000), LH (*p* = 0.000)]. **(B)** Analysis of the maximum contact area of the four paws in the three groups [*n* = 22/group, normal *vs.* veh (*), RF (*p* = 0.001), RH (*p* = 0.000), LF (*p* = 0.000), LH (*p* = 0.000)]. **(C)** Analysis of the mean intensity of the four paws in the three groups [*n* = 22/group, normal *vs.* veh (*), RF (*p* = 0.000), RH (*p* = 0.000), LF (*p* = 0.000), LH (*p* = 0.000)]. RF: right front paw; RH: right hind paw; LF: left front paw; LH: left hind paw; ***p* < 0.01, ****p* < 0.001.
