# Supplementary figures and images for: Alpha1-antitrypsin protects the immature mouse brain following hypoxic-ischemic injury
Source: Front Cell Neurosci. 2023 Mar 6;17:1137497. doi: 10.3389/fncel.2023.1137497 (PMC10025360; doi:10.3389/fncel.2023.1137497)

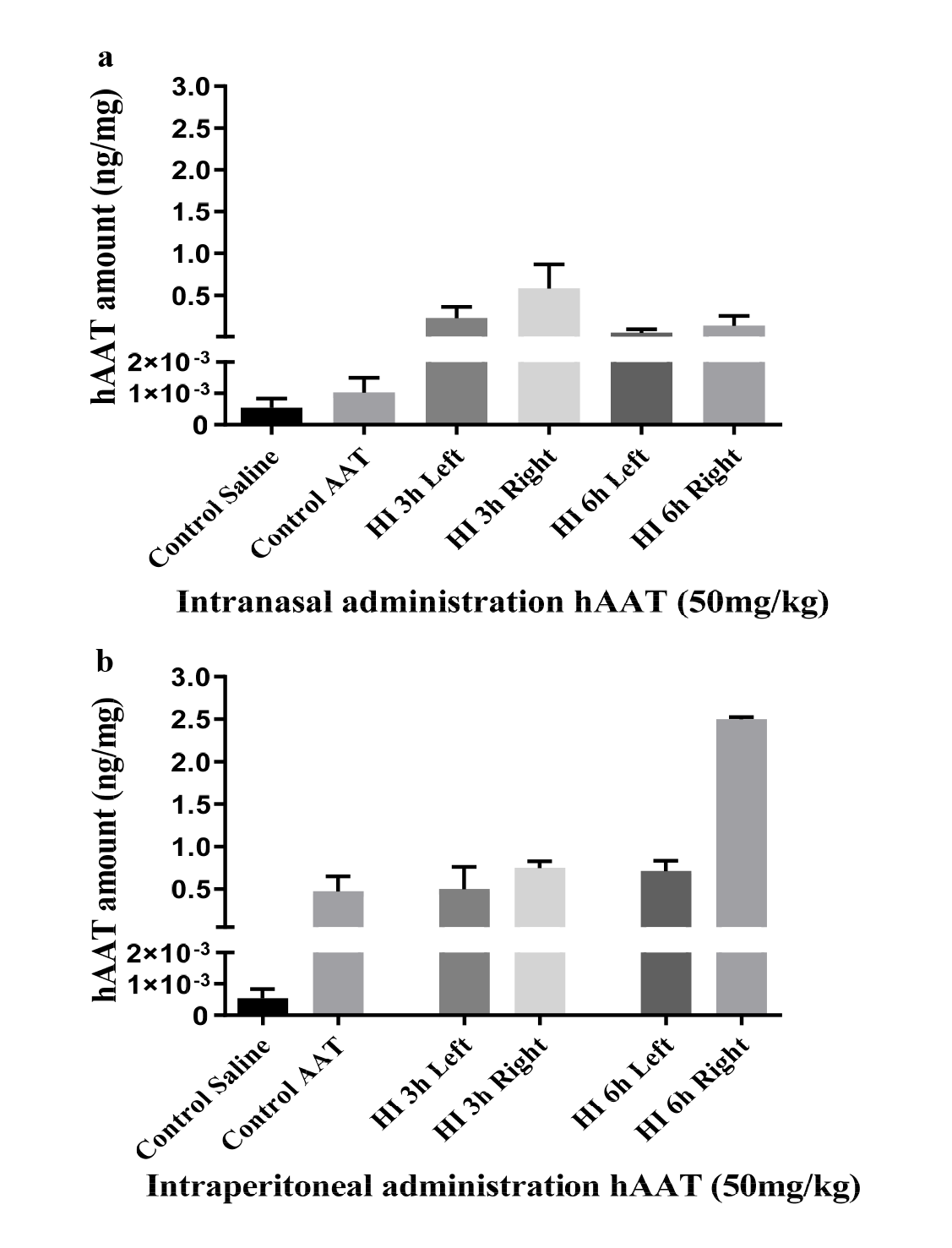

Supplement: Supplementary Figure 1 — AAT concentration in the brain by different administration routes. (A) AAT concentration in brain tissue at 3 h and 6 h after intranasal administration in the control and HI groups. (B) AAT concentration in brain tissue at 3 h and 6 h after intraperitoneal administration in the control and HI groups. [file Image_1.TIF]

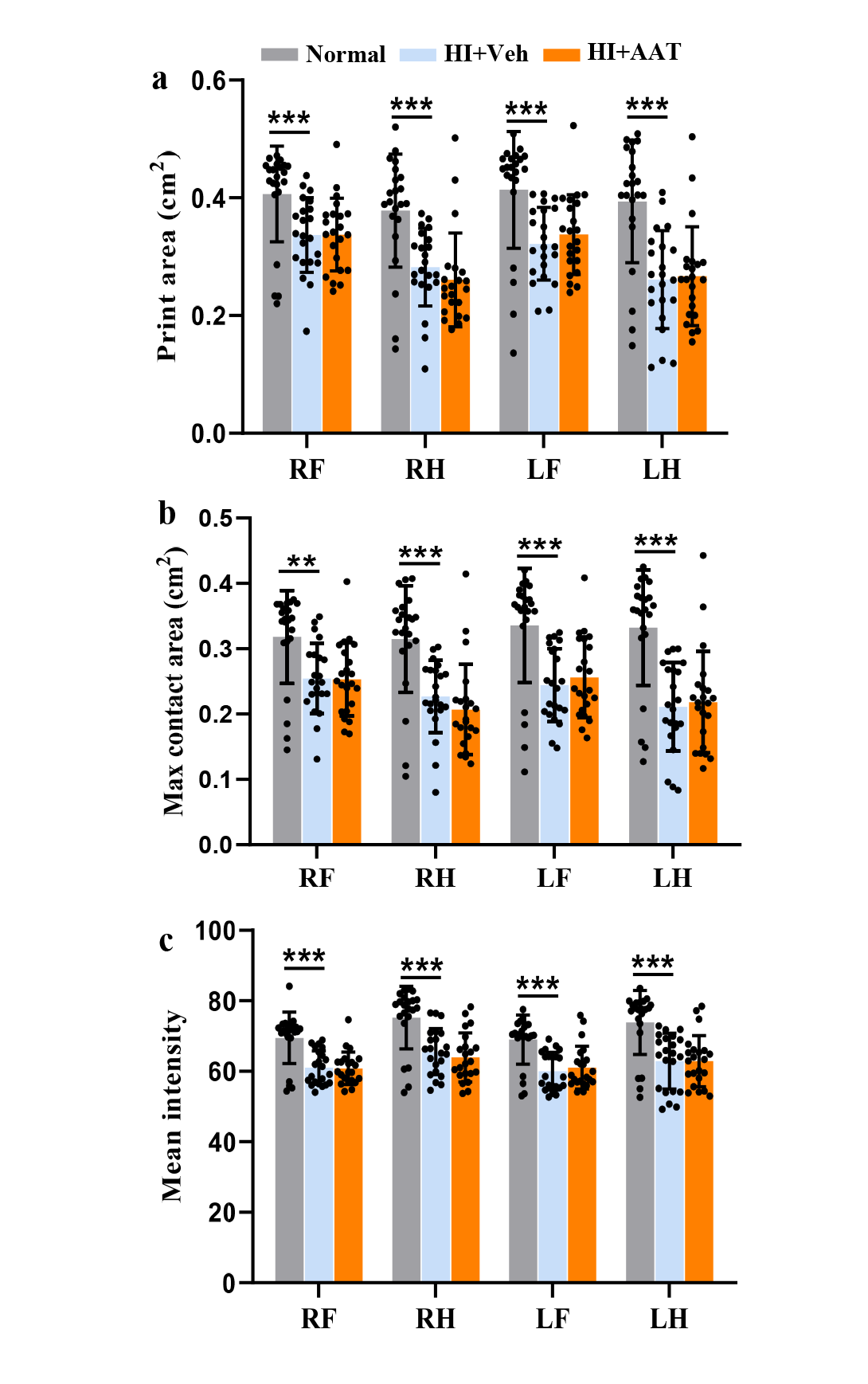

Supplement: Supplementary Figure 2 — CatWalk XT gait analysis. (A) Analysis of print area of the four paws in the normal, vehicle-treated, and AAT-treated groups [n = 22/group, normal vs. veh (*), RF (p = 0.000), RH (p = 0.000), LF (p = 0.000), LH (p = 0.000)]. (B) Analysis of the maximum contact area of the four paws in the three groups [n = 22/group, normal vs. veh (*), RF (p = 0.001), RH (p = 0.000), LF (p = 0.000), LH (p = 0.000)]. (C) Analysis of the mean intensity of the four paws in the three groups [n = 22/group, normal vs. veh (*), RF (p = 0.000), RH (p = 0.000), LF (p = 0.000), LH (p = 0.000)]. RF: right front paw; RH: right hind paw; LF: left front paw; LH: left hind paw; **p < 0.01, ***p < 0.001. [file Image_2.TIF]
